# Work Stressors and Asthma in Female and Male US Workers: Findings From the National Health Interview Survey

**DOI:** 10.1002/ajim.23722

**Published:** 2025-04-13

**Authors:** Adrian Loerbroks, Haiou Yang, Jos A. Bosch, Julia Salandi, Stefanie Keymel, Jian Li

**Affiliations:** ^1^ Institute of Occupational, Social and Environmental Medicine, Centre for Health and Society, Medical Faculty and University Hospital Düsseldorf Heinrich‐Heine‐University Düsseldorf Germany; ^2^ Center for Occupational and Environmental Health University of California Irvine California USA; ^3^ Department of Medical Psychology Amsterdam University Medical Centers Amsterdam the Netherlands; ^4^ Department of Psychology University of Amsterdam Amsterdam the Netherlands; ^5^ Department of Cardiology, Pneumology and Angiology, Medical Faculty University Hospital Düsseldorf Düsseldorf Germany; ^6^ Departments of Environmental Health Sciences and Epidemiology, Fielding School of Public Health, School of Nursing University of California Los Angeles Los Angeles California USA

**Keywords:** asthma, epidemiology, job insecurity, occupational stress, USA, work–family conflict, workplace bullying

## Abstract

**Background:**

Prior work has linked work stressors to asthma. However, research related to gender‐specific associations remains sparse and yielded mixed results. We aimed to address this gap.

**Methods:**

We drew on cross‐sectional data from the 2015 National Health Interview Survey (individual‐level response rate = 79.7%). Included were participants in employment who were aged 18–70 (*n* = 18,701). Work‐to‐family conflict, workplace bullying, and job insecurity were assessed as work stressors. Asthma was defined based on self‐reports of a lifetime diagnosis by a doctor or other health professional. To account for the complex sampling design, variance estimation was used to compute weighted descriptive statistics and odds ratios (ORs) as well as corresponding 95% confidence intervals (CIs) using multivariable logistic regression. To test for interaction, interaction terms for work stressors and gender were included in additional models.

**Results:**

In the full sample, work‐to‐family conflict, workplace bullying and job insecurity showed positive associations with asthma (OR = 1.20, 95%CI = 1.03–1.40; OR = 1.45, 95%CI = 1.17–1.80; and OR = 1.20, 95%CI = 0.99–1.45, respectively). We did not observe meaningful gender differences in the magnitudes of the ORs. All interaction terms were not statistically significant.

**Conclusions:**

Work stressors were positively associated with asthma, but there was no evidence of gender differences. Prospective studies are needed to determine the potential temporal relation of these associations.

## 1 Introduction

Work stress is common in Western countries [1] and has been found to predict a range of poor health outcomes. These include, amongst others, coronary heart disease [2], depression [2], absenteeism [3], and mortality [4]. Numerous studies – mostly carried out among European populations – have examined the link between work stressors and asthma. Those studies have nearly uniformly demonstrated positive associations of moderate magnitude [5, 6, 7, 8, 9, 10, 11, 12], although with some exceptions [13, 14, 15]. This heterogeneity may partly stem from work stress‐related gender differences: women and men may differ in (i) their degree of exposure to work stressors [16], (ii) their perception of work stressors [17, 18], and (iii) their health outcomes as responses [19, 20]. Nevertheless, gender‐specific research into the link between work stressors and asthma remains limited [5, 9, 12, 15, 21], and findings appear inconsistent: Associations have been found to be limited to men [12, 15], or only to women [21] or there was no evidence of gender‐specific associations [9]. Our aim is therefore to contribute additional evidence related to the link between work stressors and asthma by gender and to do so based on a data set representative of the US population.

Stress may exert effects on several levels (e.g., behavioral, cognitive, emotional, social, physiological), and a uniform definition or a gold standard to measure stress is lacking [22]. For instance, stress can be measured by markers of physiological arousal (e.g., cortisol), reports of stress perceptions or mood states, or reports of perturbing events such as major life events (e.g., unemployment or death of a family member) or everyday hassles [22]. Such assessments may reflect multiple domains of life or a single domain (e.g., family, work). The present study is an example of the latter, and focused on working conditions that may elicit feelings of stress in exposed workers and that we denote throughout as “workplace stressors.” Theoretical work stress models have been developed that categorize work stressors into overarching factors. The two most extensively examined models are the effort‐reward‐imbalance (ERI) model [23] and the job‐demand control model [24]. The ERI model, for example, builds on the notion that employment contracts are based on norms of social reciprocity, whereby efforts are expected to be reciprocated by adequate rewards. In the ERI model, the reward component, for instance, comprises work stressors related to the perception of one's salary, the received recognition, and promotion prospects. However, many important work stressors are not directly encompassed under such theoretical models and can be measured independently. In the current study, we drew on a data set providing information on three such work stressors, namely, work–family conflict, workplace bullying, and job insecurity. Work–family conflict refers to situations when demands associated with one's roles in work and family life are perceived as incompatible [25] (e.g., role of the employee vs. that of the parent). Workplace bullying describes situations where an individual is exposed to repeated and prolonged harassment by colleagues, supervisors, or subordinates whilst feeling unable to defend oneself [26]. Finally, job insecurity may be defined as “a perceived threat to the continuity and stability of employment as it is currently experienced” [27].

## 2 Materials and Methods

### 2.1 Study Population

We drew on data from the 2015 National Health Interview Survey (NHIS) and the 2015 Occupational Health Supplementary Survey (NHIS‐OHS) for our study. Specifically, we used the public use files, which were approved by the National Human Subjects Protection Advisory Committee. The NHIS is a cross‐sectional health survey among the general population in the US designed to produce nationally representative estimates. Recruitment is based on multistage area probability sampling and data are collected by means of face‐to‐face interviews. Before the interviewer's visit, an initial letter is sent containing information about the purpose of the NHIS, the amount of time the interview will require, and emphasizing the voluntary nature of the survey. A copy of that letter is provided to each respondent by the interviewer to obtain verbal consent for survey participation.

The NHIS consists of a core questionnaire, which remains the same across annual surveys, and a supplementary questionnaire, which varies from year to year. The core questionnaire collects demographic and health‐related data from all members of the households and one adult is selected to take part in the supplementary interview, which gathers additional data covering special health topics. Occupational health was addressed by one of the supplementary surveys that was added to the 2015 NHIS. The data set used for this study was constructed by merging the core data set and the NHIS‐OHS supplementary survey data. The individual‐level response rate was 79.7%.

In total, 33,672 adults participated in both the 2015 NHIS core survey and the NHIS‐OHS. We defined our study population based on two criteria: the first criterion was an age of 18–70 years and its application resulted in an exclusion of *n* = 5,275 who were aged 71 and above. The second criterion which was then applied was self‐reported current employment, which led to the exclusion of *n* = 9,681 who were not employed and further *n* = 15 who did not provide solid information on employment. Thus, in total, data from 18,701 individuals was available for our analyses.

### 2.2 Assessment of Study Variables

#### 2.2.1 Work‐Related Stressors

The work‐related stressors assessed in this study included work‐to‐family conflict, workplace bullying, and job insecurity. Work‐to‐family conflict was measured by the question: “Please tell me whether you: strongly agree, agree, disagree, or strongly disagree with this statement: the demands of my job interfere with my personal or family life.” “Strongly agree” and “agree” were defined as work‐to‐family conflict. Workplace bullying was measured by the item: “During the past 12 months, were you threatened, bullied, or harassed by anyone while you were on the job?” Affirmative responses were defined as exposure to workplace bullying. Job insecurity was measured by the question: “Are you worried about losing your [current/main job]?,” “Yes” was defined as job insecurity.

#### 2.2.2 Asthma Outcomes

Lifetime asthma was assessed by the item “Have you ever been told by a doctor or other health professional that you had asthma?” To assess “current asthma,” participants answering “yes” to the lifetime asthma question were then asked “Do you still have asthma?” (Response options: Yes/No). However, the latter item does not represent an established approach to define current asthma in the field of respiratory epidemiology. It remains elusive what an affirmative response captures (e.g., mild symptoms, severe symptoms, or health care utilization?). We therefore decided not to use this variable.

#### 2.2.3 Confounders

We considered the following sociodemographic variables and health‐related behaviors:

- Age, categorized into six age groups: (a) 18–24, (b) 25–34, (c) 35–44, (d) 45–54, (e) 55–64, and (f) 65–70 years.
- Sex with the response options male or female.
- Ethnicity was recorded through a question on Hispanic origin or ancestry, which was coded into the following four categories: (a) Hispanic, (b) non‐Hispanic White, (c) non‐Hispanic Black, and (d) non‐Hispanic Asian and non‐Hispanic Others.
- Education was measured by five categories: (a) less than high school, (b) high school, (c) some college, (d) college, and (e) master and above.
- Gross income, which was defined as personal yearly earnings coded into five categories, (a) under $15,000, (b) $15,000–$24,999, (c) $25,000–$44,999, (d) $45,000–$74,999, (e) $75,000 and above.
- Smoking status was defined as current, former, and never smokers. Definitions were based on two items, these were, reports of (i) having smoked at least 100 cigarettes in one's lifetime and (ii) current smoking (every day or some days). Those with negative responses on both items were defined as never smokers. Former smoking was defined as having smoked at least 100 cigarettes, but no current smoking at the time of the interview. Current smokers were defined by affirmative responses on both items.
- Obesity was defined as a body mass index (BMI) of 30 or above. The BMI was calculated based on respondents' self‐reported height (in m) and weight (in kg).

### 2.3 Statistical Analyses

For the present article, we estimated the association between every type of work stressor and lifetime asthma by separate statistical models. First, we ran unadjusted models in the entire sample and, second, corrected for age and gender. Third, we ran multivariable analyses by adjusting the models additionally for ethnicity, education, income, smoking, and obesity. Next, we ran multivariable analyses stratified by gender. To account for the complex sampling design that involves stratification and clustering of the NHIS, we used the variance estimation method in the analysis, which helps to determine the statistical reliability of descriptive statistics and measures of associations. The analysis with the variance estimation method was done using Stata 12. Associations were estimated by logistic regression models producing odds ratios (ORs) and corresponding 95% confidence intervals (CIs). Interactions of work stressors and gender were examined by including interaction terms in additional multivariable models. Individuals with missing data were excluded from the analyses.

## 3 Results

Table 1 shows the characteristics of the study population. In the full sample, 11.99% reported that they had ever been diagnosed with asthma. Among the work‐related stressors, work‐to‐family conflict was most common (25.76%) compared to workplace bullying (7.17%) and job insecurity (11.29%), but there were no striking gender differences. Most female and male participants were middle‐aged (i.e., 35–54 years), and half of the sample was female. Two‐thirds classified themselves as non‐Hispanic white, and these numbers were comparable in women and men. More than half of the sample reported to have completed college or to hold at least a master's degree and about half of the participants reported a gross income in the range of $25,000 to less than $75,000. While educational levels seemed to be largely comparable between genders, income levels were lower among women compared to men. As much as 29.57% were categorized as obese, and 14.87% were current smokers. These prevalences were similar in both genders.

**Table 1:** Description of the study sample (*n *= 18,701).

	Full sample	Male	Female
Characteristics	Weighted percent	Weighted percent	Weighted percent
**Asthma outcomes**			
Lifetime asthma			
No	88.01	89.79	86.18
Yes	11.99	10.21	13.82
**Work‐related stressors**			
Work‐to‐family conflict			
No	74.24	72.40	76.12
Yes	25.76	27.60	23.88
Workplace bullying			
No	92.83	94.18	91.45
Yes	7.17	5.82	8.55
Job insecurity			
No	88.71	88.50	88.92
Yes	11.29	11.50	11.08
**Sociodemographics**			
Age group			
18–34	34.22	34.37	34.05
35–54	43.97	44.89	43.03
55–70	21.81	20.74	22.91
Sex			
Male	50.57		
Female	49.43		
Ethnicity			
Non‐Hispanic White	67.50	68.21	66.77
Non‐Hispanic Black	12.11	9.88	14.38
Hispanic	14.13	15.23	13.01
Non‐Hispanic Asian and others	6.27	6.68	5.84
Education			
Less than high school	7.44	8.80	6.05
High school	20.50	22.57	18.38
Some college	19.34	19.04	19.64
College	37.94	36.37	39.55
Master and above	14.79	13.22	16.38
Income			
< $15,000	15.40	11.30	19.64
$15,000–$24,999	14.26	11.69	16.91
$25,000–< $44,999	26.80	25.57	28.08
$45,000–$74,999	23.78	25.56	21.93
> =$75,000	19.76	25.87	13.44
**Health‐related lifestyle factors**			
Obesity			
No	70.43	69.59	71.33
Yes	29.57	30.41	28.67
Smoking			
Never	66.64	63.84	69.80
Former	18.49	20.36	16.38
Current	14.87	15.80	13.82

In multivariable analyses (see Table 2) controlling for sociodemographics and health behavior‐related factors work‐to‐family conflict, workplace bullying and job insecurity showed positive associations with asthma in the full sample (OR = 1.20, 95%CI = 1.03–1.40, OR = 1.45, 95%CI = 1.17–1.80, and OR = 1.20, 95%CI = 0.99–1.45, respectively). We did not observe meaningful gender differences in the magnitudes of the ORs. Also, all interaction terms were statistically nonsignificant (see Table 2).

**Table 2:** Associations of work‐related stressors and lifetime asthma in the 2015 National Health Interview Survey.

	Unadjusted		Age‐ and‐sex‐adjusted		Multivariable‐adjusted					
	Full sample (*n* = 18,701)		Full sample (*n* = 18,701)		Full sample^a^ (*n* = 16,140)		Men^b^ (*n* = 7967)		Women^b^ (*n* = 8173)	
	OR	95% CI	OR	95% CI	OR	95% CI	OR	95% CI	OR	95% CI
Work‐to‐family conflict	1.16	1.02–1.33	1.20	1.05–1.37	1.20	1.03–1.40	1.14	0.90–1.44	1.26	1.03–1.53
Workplace bullying	1.68	1.36–2.07	1.63	1.32–2.02	1.45	1.17–1.80	1.40	0.95–2.06	1.48	1.13–1.93
Job insecurity	1.20	1.01–1.42	1.25	1.05–1.48	1.20	0.99–1.45	1.27	0.95–1.70	1.13	0.86–1.48

The *p*‐values for interaction terms for the respective work stressor and gender in the multivariable models were as follows:

Work‐to‐family conflict * gender *p* = 0.36.

Workplace bullying * gender *p* = 0.72.

Job insecurity * gender *p* = 0.86.

Abbreviations: CI = confidence interval; OR = odds ratio.

^a^ Adjusted for age, gender, ethnicity, education, income, smoking, and obesity.

^b^ Adjusted for age, ethnicity, education, income, smoking, and obesity.

## 4 Discussion

In the present study, work‐to‐family conflict, workplace bullying, and job insecurity were associated with increased odds of asthma. We did not observe gender differences though.

### 4.1 Findings in Light of Prior Research

As mentioned above, work–family conflict occurs when the demands in one's work life and in family life are perceived to interfere [25]. In this respect, it needs mentioning that work–family conflicts can be bidirectional and that therefore a distinction can be made between demands at work that interfere with family life (referred to as “work‐to‐family conflict”) and demands related to the family that interfere with meeting the demands at work (i.e., “family‐to‐work conflict”) [25, 28]. In a prior cross‐sectional study on work stress and family stress among women in China, work stress and family stress were each positively associated with asthma and their combined exposure was associated with an excess of asthma occurrence [11]. While this approach considers the combined exposure, it fails to cover work–family conflict as the experience of *conflicting* demands in both domains in life. In this respect, the present study provides novel evidence. Additional research is needed, however, in this field (see below). Workplace bullying was previously examined in relation to asthma in a prior study among cleaners (64.1% female) and a control sample of workers in Peru [10]. In that study, Radon et al. [10] found a strong positive association of workplace bullying with the odds of asthma. Bullying was assessed by three items that were combined into a summary score and that covered communication problems, personal discredit, and threats at work during the 12 months before the survey. We used the 2015 NHIS data in our study, but the link between workplace bullying and a broad range of outcomes (including asthma) has also been addressed based on the NHIS data collected in 2010 [12]. That latter study examined associations by gender and reported a link between workplace bullying and asthma in men but not in women [12]. By contrast, we did not observe gender differences based on our data. Our findings of a positive association of job insecurity with asthma are consistent with prior studies that linked job insecurity to an elevated asthma prevalence [14] or incidence [8]. Our study adds to this evidence in addressing gender differences.

As to the biological plausibility of our findings, there is ample evidence supporting the notion that psychological stress affects asthma [29, 30]. Two key biopsychological pathways that translate psychological stress into physical stress responses include the sympathetic‐adreno‐medullary nervous system and the hypothalamic‐pituitary‐adrenal axis [31]. Repeated stress exposures have been associated with reduced expression of the genes encoding the glucocorticoid and the β_2_‐adrenergic receptor, which in turn may reduce the response to inhaled corticosteroids and β_2_‐agonists [30, 32, 33]. In parallel, these stress‐related endocrine disruptions, exert potent immunomodulatory effects and may translate into an atopy (or Th_2_)‐biased response [34], which biases immune reactions toward allergic (e.g., mast‐cell and IgE‐mediated) hypersensitivity responses [35].

### 4.2 Strengths and Weaknesses

Strengths of our study include its sample: first, our sample can be assumed to be representative for the US population and, second, its size seems to provide sufficient statistical power to examine gender‐specific associations. The current study also has a number of drawbacks. First, the NHIS is a cross‐sectional study and such designs are unable to disentangle the potential directions which may underlie the observed associations. For instance, we cannot rule out that a sense of job insecurity develops as a result of living with asthma, especially if it is poorly controlled: prospective studies have shown that asthma is associated with poorer employability (e.g., increased risk of absenteeism [36], work disability [37], and exit from full‐time employment) and one may speculate that those outcomes are preceded by a sense of job insecurity.

Second, we measured the work stressors by single items. More detailed measures might have improved our understanding of the potential relationships. For instance, we assessed to what extent work‐related obligations interfere with family life, but not to what extent family life is felt to impair working life. With regard to workplace bullying, self‐labeling as a victim of bullying based on a single item, as in our study, has shown its utility in previous research [26, 38, 39]. However, the item in the present study (“During the past 12 months, were you threatened, bullied, or harassed by anyone while you were on the job?”) lacked some defining elements of bullying, such as the persistent exposure or victims' perception of powerlessness [26]. Furthermore, data on the perpetrators (e.g., clients, colleagues, or supervisors) had been of interest. It would also be worthwhile to measure workplace bullying not only in self‐labeling as a victim, but also based on the exposure to bullying behavior [26]. While a universally accepted definition of job insecurity is lacking, three defining characteristics seem to have consensus: (i) the subjective experience (in contrast to markers of objective insecurity, such as insecure contracts), (ii) the expectation of an event in the future, (iii) the threat to one's current job [27]. The item we used in this study seems to capture those elements (i.e., “Are you worried about losing your [current/main job]?”). Although we have been unable to fully capture all nuances of the work stressor concepts addressed in our study, this does not imply that single‐item measures are invalid. In fact, the satisfactory concurrent and predictive validity of single‐item work stressor measures is well documented [40]. If our dichotomous work stressor items were limited in validity, respondents would have been misclassified into incorrect response options (so‐called misclassification). Such misclassification would affect our association measures only if it was related to self‐reports of asthma (i.e., differential misclassification). It is difficult to envisage that the accuracy of reporting work stressors would depend on one's asthma status; therefore, we assume that any misclassification is non‐differential, which would likely attenuate the observed associations. Consequently, the relationships between work stressors and asthma may have been underestimated in our study.

Third, we relied on self‐reported information to define asthma in contrast to clinical data (e.g., spirometry). Self‐report data are often the only feasible option to determine asthma in large epidemiological studies [41]. Those self‐reports may rely on characteristic asthma symptoms (e.g., wheezing) or on reports of a prior diagnosis of asthma (lifetime asthma) [41, 42]. In the NHIS, lifetime asthma was measured by an item addressing the prior diagnosis of asthma by a doctor or other health professional. Self‐reports of lifetime asthma diagnoses have found to be reliable (i.e., diagnosed by a physician or nurse) [43]. Further, such reports show good agreement with administrative health data and do so irrespectively of whether the diagnoses were reported to be established by a physician or not [44]. It is also encouraging for this study's validity that the lifetime prevalence of self‐reported asthma in our study (12.0%) is consistent with other estimations: in the Behavioral Risk Factor Surveillance System survey, which used a similar questionnaire item, the asthma prevalence in 2015 in the US was estimated at 13.8% [45].

Fourth, while we adjusted our analyses for potentially important confounders, we failed to consider occupational exposures in particular: asthma exacerbations can be triggered by a large number of agents in the workplace (e.g., dust, chemical fumes, mold, secondhand smoke, physical activity). It seems plausible that many of the workplaces which are characterized by exposure to such asthma triggers are also those with poorer psychosocial working conditions (i.e., job insecurity). In fact, in some professions, multiple of those agents may be present at the same time (e.g., cleaning staff or waiters). Notably, though, in a prior study [7], we have examined the link between work stress and the risk of asthma and were able to adjust our estimates for such occupational asthma risk. To do so, we used a variable that collapsed information of self‐reported exposure to chemicals, or to heat, cold or moisture, or employment in an asthma‐risk profession. Adjustment for this variable in the prior study changed our estimates only marginally which demonstrated limited potential for confounding. This may also apply for the current study. However, the scope of considered agents was very limited and broader assessment is recommended in future studies on work stressors and asthma. In addition, some confounders may not have been measured in desirable detail (e.g., current smoking: it remains unclear for how long respondents have smoked daily), and those confounders may have partially affected our findings despite statistical adjustment (so‐called residual confounding).

A final limitation is that our data stem from 2015. It remains elusive to what extent our findings can be generalized to the post‐Covid‐19 era, which has affected working life and (gender‐specific) career prospects in the US [46] and elsewhere. In most European countries, for instance, home office work has become more common in post‐pandemic working life [47]. The same holds true ‐ to a more limited extent – to flexibility in working time [47]. Remote work has been associated with less work–family conflict, especially in women [48], and also with less bullying [49].

In conclusion, we found work‐to‐family conflict, workplace bullying, and job insecurity to be related to asthma but did not observe gender‐specific associations. To corroborate our findings, research is needed that builds on (1) prospective observational study designs, (2) more extensive assessments of the work stressors we addressed, (3) data from the post‐Covid‐19‐era, and (4) sufficiently powered gender‐specific analyses.

## Author Contributions

Jian Li conceived the idea of using the National Health Interview Survey data. Haiou Yang carried out the statistical analyses. Adrian Loerbroks prepared a first draft of the manuscript. All authors contributed substantially to the interpretation of the data and to the revision of the manuscript for important intellectual content.

## Disclosure

John Meyer declares that he has no conflict of interest in the review and publication decision regarding this article.

## Ethics Statement

We used public‐use files approved by the National Human Subjects Protection Advisory Committee. We did not obtain specific ethical approval to use the National Health Interview Survey public use files.

## Conflicts of Interest

The authors declare no conflicts of interest.

## Data Availability

The authors have nothing to report.
